# Surveillance for feline herpesvirus type 1 mutation and development of resistance in cats treated with antiviral medications

**DOI:** 10.3389/fvets.2023.1197249

**Published:** 2023-05-18

**Authors:** Andrew C. Lewin, Nikole E. Ineck, Melanie A. Mironovich, Morgan E. Marino, Chin-Chi Liu, Ugochi Emelogu, Erinn P. Mills, Pilar Camacho-Luna, Renee T. Carter

**Affiliations:** Department of Veterinary Clinical Sciences, School of Veterinary Medicine, Louisiana State University, Baton Rouge, LA, United States

**Keywords:** feline herpesvirus, cidofovir, famciclovir, ganciclovir, antiviral, resistance, mutation, genomics

## Abstract

Feline herpesvirus type 1 (FHV-1) commonly causes ocular surface disease in cats and is treated with antiviral medications targeting viral DNA polymerase (UL30/42). Herein, we describe a method to assess the FHV-1 genome for mutation development and to assess the functional impact of mutations, if present. Fourteen shelter-housed domestic cats with FHV-1 ocular surface disease were assigned to one of four treatment groups: placebo (*n* = 3), cidofovir 0.5% ophthalmic solution (*n* = 3), famciclovir oral solution (*n* = 5), or ganciclovir 0.15% ophthalmic solution (*n* = 3). Swabs were collected before (day 1) and after (day 8) 1 week of twice-daily treatments to isolate viable FHV-1. Viral DNA was extracted for sequencing using Illumina MiSeq with subsequent genomic variant detection between paired day 1 and day 8 isolates. Plaque reduction assay was performed on paired isolates demonstrating non-synonymous variants. A total of 171 synonymous and 3 non-synonymous variants were identified in day 8 isolates. No variants were detected in viral UL23, UL30, or UL42 genes. Variant totals were not statistically different in animals receiving antiviral or placebo (*p* = 0.4997). A day 8 isolate from each antiviral treatment group contained a single non-synonymous variant in ICP4 (transcriptional regulator). These 3 isolates demonstrated no evidence of functional antiviral resistance when IC_50_ was assessed. Most (10/14 pairs) day 1 and 8 viral isolate pairs from the same host animal were near-identical. While functional variants were not detected in this small sample, these techniques can be replicated to assess FHV-1 isolates suspected of having developed resistance to antiviral medications.

## 1. Introduction

Feline herpesvirus type 1 (FHV-1) is a double stranded DNA virus with a narrow host range, including domestic cats (1, 2) and wild felids (3). Previous assessments of the FHV-1 genome have confirmed that FHV-1 demonstrates relatively low viral genomic diversity (1, 3–5) and that host disease severity is unlikely to be related to viral genome variants (6). FHV-1 in domestic cats is frequently treated with antiviral medications targeting viral genes responsible for viral replication (UL23, UL30, and UL42). Numerous antiviral medications have previously been assessed for treatment of FHV-1 related disease in cats, including famciclovir (the prodrug of penciclovir) (7–12), cidofovir (12, 13), and ganciclovir (12, 14, 15), amongst others. All 3 of these drugs are commonly used for treatment of FHV-1 in cats, and act through inhibition of viral DNA polymerase (7).

Herpes simplex virus (HSV-1/2) is an alphaherpesvirus (like FHV-1) which causes disease in human hosts. HSV-1/2 resistance to antiviral medications has been extensively documented (16–19) and occurs predominantly in immunocompromised individuals treated over relatively long periods with antiviral medications (19–21). In contrast, FHV-1 resistance to antiviral medications has not been previously documented or assessed in depth, to the authors’ knowledge. Rapid identification of viral genome mutation is possible using high-throughput full viral genome sequencing, with functional alterations confirmed using traditional *in-vitro* testing. The objective of the present study was to detail a practical method to screen FHV-1 genomes for mutation development and to assess the functional impact of these mutations, if present. We hypothesized that we would not detect clinically significant FHV-1 genome mutations and development of functional antiviral resistance in this small sample of FHV-1 isolates, based on sample size and previous assessments demonstrating that the FHV-1 genome is highly conserved (1, 3, 5, 6).

## 2. Methods

### 2.1. Host animals, drug administration, and sample acquisition

Domestic cats enrolled in a recent clinical trial comparing three treatments for naturally-acquired ocular FHV-1 (12) were utilized in the present study. All procedures were performed in accordance with an approved Louisiana State University Institutional Animal Care and Use Committee protocol (LSU 19–034). All cats were housed in animal shelter facilities at the time of sampling and had ocular surface disease consistent with FHV-1 infection. Quantitative ocular disease scoring was performed by a trained observer, as previously described (12). All cats were confirmed to be actively shedding FHV-1 using quantitative polymerase chain reaction (qPCR) (Texas A&M Veterinary Medical Diagnostic Laboratory). On entry to the shelter, cats were screened for feline immunodeficiency virus and feline leukemia virus (SNAP, Idexx). Cats were randomly allocated to receive one of four treatment protocols: ophthalmic placebo solution (eyewash) + oral lactulose placebo, cidofovir 0.5% ophthalmic solution + oral lactulose placebo, ophthalmic placebo solution (eyewash) + famciclovir oral suspension (250 mg/mL at 90 mg/kg), or ganciclovir 0.15% ophthalmic solution + oral lactulose placebo. Shelter staff were masked to treatment group for all animals. All cats received one ocular preparation and one oral preparation twice daily regardless of group. All medications and placebo were acquired from compounding (503A and 503B) facilities (Stokes Pharmacy and Epicur Pharma) with drug concentrations validated at an independent external laboratory. All cats were sampled before (day 1) and after (day 8) 7 days of treatment using swabs (sterile foam tipped swab, Puritan) rolled against the oropharyngeal and conjunctival surfaces. Following sample acquisition, each swab was immediately placed into viral transport media (composed of Hank’s balanced salt solution, heat-inactivated fetal bovine serum, gentamicin sulfate and amphotericin B, Thermo Fisher) and placed on ice for transportation.

### 2.2. Viral isolation and DNA extraction

On the same day as sample collection, viral isolation was performed using Crandell Rees feline kidney cells (CRFK) (ATCC), as previously described (1, 3). Cells were cultured in Dulbecco’s modified Eagle medium (DMEM; Life Technologies) with 10% fetal bovine serum (VWR) and 1% penicillin and streptomycin sulfate (Life Technologies) at 37°C/5% CO2. A 0.5 mL aliquot of each sample was added to individual T25 culture flasks containing maximally confluent CRFK cells along with 2 mL of DMEM containing 2% fetal bovine serum and 1% penicillin and streptomycin before being placed on a rocker for 60 min. An additional 4 mL of DMEM was then added to each flask before being incubated at 37°C with 5% CO_2_ and checked daily until 100% cytopathic effect (CPE) was observed. The infected cells and media in the flask were then frozen at −80°C and thawed at room temperature for 3 cycles. The sample was centrifuged at 1,000 rpm for 5 min (ThermoScientific Heraeus Megafuge 8R centrifuge) to acquire supernatant which was stored at −80°C.

Viral DNA extracted from the frozen supernatant was prepared using a commercial kit according to the manufacturer’s instructions (PureLink Viral RNA/DNA Mini Kit), as previously described (3). DNA purity and concentration were assessed using a Qubit dsDNA HS Assay Kit (Life Technologies). Samples were confirmed to contain FHV-1 DNA prior to sequencing using qPCR (ABI 7900-2) with FHV-1 thymidine kinase primers (22) (IDT).

### 2.3. Full viral genome sequencing

Viral genome sequencing was performed as previously described (3, 23). DNA samples were diluted to 1 ng DNA in 5 mL solution and libraries were constructed according to the Nextera XT DNA Library Preparation Kit protocol (FC-131-1096; Illumina Inc.). Libraries were amplified and indexed using Nextera XT Index Kit v2 (Set A; Illumina Inc.). Quality and quantity of finished libraries were assessed using a Fragment Analyzer Instrument (Advanced Analytical) and dsDNA HS Assay Kit. Indexed libraries were pooled and paired-end sequenced using an Illumina MiSeq 500 bp (v2) sequencing kit (MS-102-2003).

### 2.4. Viral genome assembly and alignment

Reference-based assembly was performed using Geneious Prime ver 2022.2.2. Paired-end reads were trimmed using BBDuk adapter/quality trimmer ver 38.84 (right end, Kmer length = 27, maximum substitutions = 1, minimum quality = 20, minimum overlap = 20, minimum length = 20). Trimmed paired-end reads were then assembled to FHV-1 reference sequence C-27 (GenBank accession NC_013590). A consensus sequence was extracted from aligned reads with gaps filled with “N’s.” Genomes were then annotated and submitted to GenBank using Geneious Prime ver 2022.2.2. Genbank accession numbers for each isolate are shown in Table 1.

**Table 1:** Host animal signalment, treatment group allocation and related FHV-1 sequence IDs.

Host animal ID	Treatment group	Age (months)	Sex	Host ocular clinical scores after 7 days treatment	Sequence ID	Study day 1 or day 8	Genbank accession number
1	Placebo	3	M	Improved	LOU-1	1	OQ756192
					LOU-2	8	OQ756193
2	Cidofovir	2	M	No change	LOU-3	1	OQ756194
					LOU-4	8	OQ756195
3	Cidofovir	2	M	Improved	LOU-5	1	OQ756196
					LOU-6	8	OQ756197
4	Famciclovir	24	M	Worsened	LOU-7	1	OQ756198
					LOU-8	8	OQ756199
5	Famciclovir	3	F	Improved	LOU-9	1	OQ756200
					LOU-10	8	OQ756201
6	Ganciclovir	24	F	Worsened	LOU-11	1	OQ756202
					LOU-12	8	OQ756203
7	Ganciclovir	12	F	Improved	LOU-13	1	OQ756204
					LOU-14	8	OQ756205
8	Placebo	2	M	Improved	LOU-15	1	OQ756206
					LOU-16	8	OQ756207
9	Placebo	3	M	Worsened	LOU-17	1	OQ756208
					LOU-18	8	OQ756209
10	Cidofovir	2	M	Improved	LOU-19	1	OQ756210
					LOU-20	8	OQ756211
11	Famciclovir	2	F	Improved	LOU-21	1	OQ756212
					LOU-22	8	OQ756213
12	Famciclovir	3	M	Worsened	LOU-23	1	OQ756214
					LOU-24	8	OQ756215
13	Famciclovir	24	F	Worsened	LOU-25	1	OQ756216
					LOU-26	8	OQ756217
14	Ganciclovir	2	F	Improved	LOU-27	1	OQ756218
					LOU-28	8	OQ756219

Host ocular clinical scores after 7 days treatment taken from Mironovich et al. (12). M, Male; F, Female.

Viral genomes were aligned using MAFFT ver 7.490 within Geneious Prime ver 2022.2.2 (24). Default parameters were used for all alignments (scoring matrix of 1PAM/k = 2, gap penalty of 1.53 and offset value of 0.123). Alignments of FHV-1 genomes were created using a canine herpesvirus type 1 (CHV-1) outgroup (0194, GenBank Accession NC_030117.1).

### 2.5. Variant detection

Analysis of variants was performed as previously described using the Geneious variant finder within Geneious Prime ver 2022.2.2 (3, 23). Each day 8 isolate was individually compared to the corresponding day 1 isolate to identify variants which had developed over the course of the 7-day treatment period. Variant totals were compared between treatment groups using one way ANOVA with significance set at *p* < 0.05.

Alignments were analyzed (1,000 bootstrap replicates) using ModelFinder (25) via IQ-Tree 2 ver 1.6.12 (26), which described the best-fit model (TVM + F + R4/5). The resultant maximum likelihood tree was visualized using Splitstree ver 4.16.1 (27).

### 2.6. Plaque reduction assay

*In-vitro* IC_50_ analysis was performed as previously described (14) on viral pairs where day 8 isolates demonstrated non-synonymous mutations detected by genome variant analysis. IC_50_ in the present study was defined as the concentration of antiviral drug at which the number of observed viral plaques is reduced by 50%, relative to untreated infected cells. CRFK cells were cultured in Dulbecco’s modified Eagle medium (DMEM; Life Technologies) with 10% fetal bovine serum (VWR) and 1% penicillin and streptomycin sulfate (Life Technologies) at 37°C/5% CO2 on 24 well plates until maximally confluent. Fifty viral plaque-forming units of each FHV-1 isolate were absorbed by the cells in each well over 1 h (on a rocker at room temperature). Based on the antiviral drug each individual host cat received, the supernatant in each well was then replaced with DMEM containing 1% methylcellulose, 2% FBS and 1% penicillin–streptomycin ± ganciclovir (Acros Organics), cidofovir (Apexbio) or penciclovir, the active metabolite of famciclovir (TCI). Plates were incubated at 37°C/5% CO2 for 48 h, then fixed with methanol and stained with 0.5% crystal violet solution (Fisher Scientific). Viral plaque numbers were then counted using a light microscope. Concentrations of each antiviral assessed were based on previously published mean IC_50_ values for FHV-1 (7): 8.9 μM for ganciclovir, 19 μM for cidofovir and 14 μM for penciclovir. Multiple concentrations of each antiviral were assessed in order to determine IC_50_ values for each day 1 and day 8 isolate; 0.25 X IC_50,_ 0.5 X IC_50,_ 1 X IC_50,_ 2 X IC_50_ and 5 X IC_50._ Positive (no antiviral) and negative controls (no virus) were included and all wells were repeated in triplicate. IC_50_ values were determined for each day 1 and day 8 isolate and values were compared for matched pairs. Antiviral resistance was considered to have developed if a 3-fold increase in IC50 was present (28) in day 8 isolates, relative to day 1 isolates.

### 2.7. *In-vitro* cellular viability analysis

To ensure that reduction in viral plaque number was unrelated to CRFK cellular viability, a cellular cytotoxicity assay was utilized (CellTiter-Glo^®^ kit, Promega) according to manufacturer recommendations. Trypsinized CRFK cells were transferred to 96-well tissue culture plates (Corning Costar) and incubated in DMEM +10% FBS/1% PS until confluent as outlined above. Growth medium was decanted and replaced with DMEM +2% FBS/1% PS containing 0.25X, 0.5X, 1X, 2X, or 5X the IC_50_ values for each antiviral compound (cidofovir, ganciclovir, and penciclovir), as utilized for the plaque reduction assay. Negative control wells containing CRFK cells in media without added antiviral compounds and separate blank wells containing only liquid media without cells were included on each plate, to allow for creation of corrected controls. Plates were incubated at 37°C/5% CO2 for 48 h and were then transferred to room temperature for 30 min. CellTiter-Glo^®^ reagent was added to each well and cell lysis was induced using an orbital shaker at room temperature for 2 min. Plates were then incubated at room temperature for 10 min before luminescence was measured with a microplate reader (Synergy HTX, BioTek). Four replicates were assessed for each antiviral concentration. Cytotoxicity was expressed as relative luminescence, calculated as the mean luminescence of a given antiviral concentration divided by the mean luminescence of the corrected plate negative control.

## 3. Results

### 3.1. Host animals and viruses

A total of 14 host animals actively shedding FHV-1 were sampled to obtain 28 isolates of FHV-1 (Table 1). The median age of the host cats was 3 months with a range of 2–24 months. A total of six females and eight male animals were sampled. Three animals received placebo medications, three received cidofovir 0.5% ophthalmic solution, five received famciclovir oral suspension (250 mg/mL at 90 mg/kg) and three received ganciclovir 0.15% ophthalmic solution. Five of the 14 animals experienced worsened ocular clinical signs following treatment, 8/14 animals improved and 1/14 had no change in ocular clinical signs (Table 1). Of the five animals which experienced worsened ocular clinical signs, 3/5 were receiving famciclovir, 1/5 was receiving ganciclovir and 1/5 was receiving placebo. All enrolled animals were seronegative for feline immunodeficiency virus and feline leukemia virus.

### 3.2. Sequencing summary

A total of 28 FHV-1 isolates (14 pairs) were fully sequenced using Illumina Miseq. The total number of reads ranged from 251,648 to 4,643,990 and the number of reads mapped to the reference genome ranged from 34,056 to 2,993,610. The mean coverage of the FHV-1 genome ranged from 56.4 to 5114.1. The GC content of the FHV-1 genomes sequenced ranged from 44.7 to 46% and genome lengths ranged from 135,996 to 140,625, which is consistent with previous reports of FHV-1 (1, 3). Individual isolate sequencing information is shown in Supplementary Table S1.

### 3.3. Variant detection

Variants were detected in all day 8 isolates when compared with the paired day 1 isolates and are summarized in Table 2. All detected variants in each day eight isolate are shown in Supplementary Table S2. No variants were detected in viral genes associated with viral replication; thymidine kinase (UL23) and DNA polymerase (UL30 and UL42). The median (range) number of genome variants was 10 (10-38) in FHV-1 from hosts receiving cidofovir, 9 (4-9) in FHV-1 from hosts receiving ganciclovir, 13 (2-27) in FHV-1 from hosts receiving famciclovir and 12 (2-15) from hosts receiving placebo. There was no significant different in variant totals by host treatment (*p* = 0.4997). The vast majority (171/174) of variants led to synonymous amino acid changes, whereas only three were non-synonymous. These were found in the day eight isolate from three separate animals receiving either famciclovir (host animal 12, LOU-23/24), cidofovir (host animal 2, LOU-3/4) or ganciclovir (host animal 14, LOU-27/28). In all three cases, the single non-synonymous variant was located in the FHV-1 ICP4 gene.

**Table 2:** Variant totals for each FHV-1 viral pair.

Host animal ID	Treatment group	Sequence ID	Total synonymous variants	Total non-synonymous variants
1	Placebo	LOU-1/2	2	0
2	Cidofovir	LOU-3/4	37	1
3	Cidofovir	LOU-5/6	10	0
4	Famciclovir	LOU-7/8	27	0
5	Famciclovir	LOU-9/10	13	0
6	Ganciclovir	LOU-11/12	9	0
7	Ganciclovir	LOU-13/14	4	0
8	Placebo	LOU-15/16	12	0
9	Placebo	LOU-17/18	15	0
10	Cidofovir	LOU-19/20	10	0
11	Famciclovir	LOU-21/22	2	0
12	Famciclovir	LOU-23/24	15	1
13	Famciclovir	LOU-25/26	7	0
14	Ganciclovir	LOU-27/28	8	1

**Figure 1:**
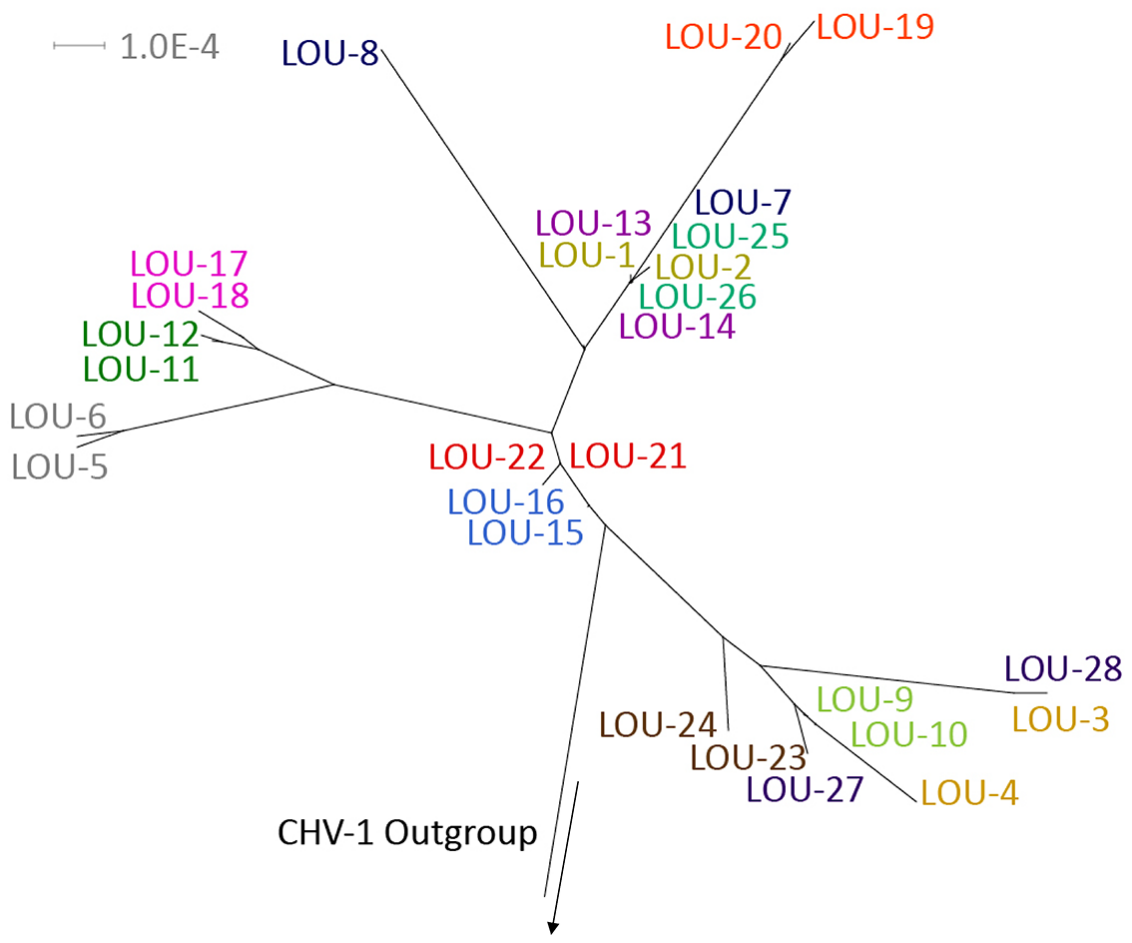
Phylogenetic tree of the 28 sequenced FHV-1 isolates with a CHV-1 outgroup. Note how the majority of viral pairs are closely clustered together, with the exception of LOU3/4, LOU-7/8, LOU-23/24, and LOU-27/28, where each day 8 isolate contained a relatively high total of variants, at least 1 non-synonymous variant or both. Made using Splitstree ver 4.16.1.

Phylogenetic tree analysis was utilized to visualize and estimate distances between pairs of isolates with a CHV-1 outgroup (Figure 1). For the majority of isolates (10/14), pairs clustered closely together. Four pairs of isolates were not clustered immediately together (LOU-3/4, LOU-7/8, LOU-23/24, and LOU-27/28). In each of these four cases, the day 8 isolate contained a relatively high total of variants (LOU-8), at least 1 non-synonymous variant (LOU-24, LOU-28) or both (LOU-4) (Table 2).

### 3.4. Plaque reduction assay and cellular viability assay

**Figure 2:**
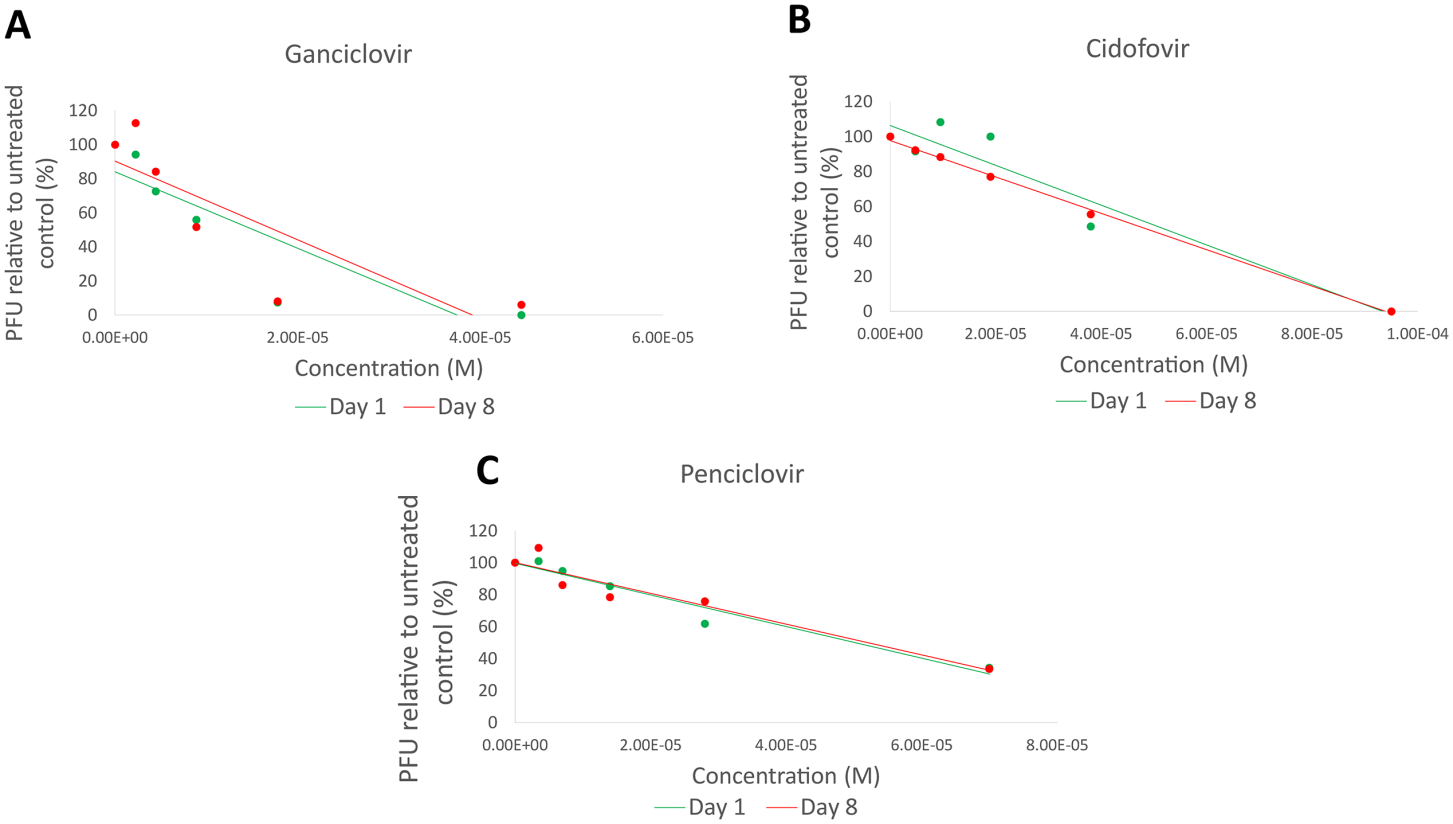
Plaque reduction assay results for 3 pairs of isolates (LOU-3/4, LOU-23/24, and LOU-27/28) which demonstrated non-synonymous variants. No evidence of functional antiviral resistance was detected: Ganciclovir (LOU-27/28) results are shown in **(A)**, cidofovir (LOU-3/4) results are shown in **(B)**, and penciclovir (LOU-23/24) results are shown in **(C)**. PFU, Plaque forming units; M, Molar.

Three pairs of isolates (LOU-3/4, LOU-23/24, and LOU-27/28) which demonstrated non-synonymous variants were chosen for *in-vitro* antiviral plaque reduction assay analysis (Table 2). These isolates were obtained from host animal 2 (treated with cidofovir), host animal 12 (treated with famciclovir) and host animal 14 (treated with ganciclovir). In summary, no evidence of antiviral drug resistance (>3 fold increase in IC_50_ values) was detected in any of the 3 day 8 isolates assessed. The IC_50_ values were 49.5 μM (day 1, LOU-3) and 45.8 μM (day 8, LOU-4) for isolates from host animal 2 (cidofovir), 50.2 μM (day 1, LOU-23) and 52.1 μM (day 8, LOU-24) for isolates from host animal 12 (penciclovir) and 15.2 μM (day 1, LOU-27) and 17.5 μM (day 8, LOU-28) for isolates from host animal 14 (ganciclovir) (Figure 2). CRFK cellular viability was maintained at all concentrations of antiviral which were assessed and visual observation of cellular monolayers confirmed this finding.

## 4. Discussion

In summary, the methods described above can be successfully utilized to screen FHV-1 isolates for development of genome variants and functional antiviral resistance. While we did not detect significant mutation and development of functional antiviral resistance in any of the isolates in the present study, this is not unexpected due to the relatively small sample size and anticipated high degree of FHV-1 genome conservation. Subtherapeutic dosing of antiviral medications for cats with FHV-1 has previously been identified as a possible mechanism for development of resistant isolates (7). While antiviral drug resistance in related viruses like HSV-1/2 (16–19) is well documented, to the authors’ knowledge, this has not been observed for FHV-1 to date. The genome of FHV-1 is highly conserved, and variants are infrequent, particularly when compared to other members of the varicellovirus genus (1, 3–5, 29, 30). Despite 90+ FHV-1 isolate genomes from at least 3 global continents being sequenced, viral variants which could confer antiviral resistance have not yet been identified.

In order to confirm functional antiviral resistance, we used a classic plaque reduction assay technique, which has been previously utilized for detection of HSV-1 resistance to helicase-primase inhibitors (31). The approach used in the present paper requires a feline cell line (due to the high species specificity of FHV-1). Otherwise, the techniques utilized for *in-vitro* confirmation of antiviral resistance are very similar to those utilized for HSV-1 (31, 32) The authors of the present study have utilized a similar technique for assessment of ganciclovir inhibition of FHV-1 replication (14). Despite not detecting antiviral resistance in any of the isolates utilized for the present study, this well-established *in-vitro* technique has been previously utilized for demonstration of functional antiviral resistance in herpesviruses, including FHV-1.

More recently, the use of High Throughput Sequencing (HTS) has received attention for the detection of variants in herpesviruses. The authors of the present study have previously utilized HTS for the detection of variants in FHV-1 (1, 3) as well as computational assessment of the relationship between FHV-1 genome variants and disease severity in feline hosts (6). The HTS sequencing and analysis techniques used in the present study are near identical to these previous works, with the exception that the reference isolate used for detection of day 8 variants in the present study was always the corresponding day 1 isolate (rather than a historical isolate obtained from a different host). In this way, isolate-specific variants which developed following host antiviral treatment could be detected, if present. Similar techniques using the same HTS platform (Illumina) to detect variants which could possibly confer antiviral resistance have been published for human herpesvirus type 6 (33) and HSV-1/2 (34). The latter of these two approaches utilized gene-specific HTS sequencing (TK and Pol), which differs from the approach utilize in the present study (whole viral genome sequencing), but overall the approach is similar.

The host animals in the present study were treated for 7 days and aside from upper respiratory tract infection, were apparently healthy. HSV-1/2 resistance to antiviral drugs predominantly occurs in immunocompromised individuals (19–21, 35) although acyclovir resistant HSV-1 keratitis can occasionally occur in immunocompetent individuals (36). Further assessment of FHV-1 from immunocompromised cats, such as those infected with FIV, is warranted. Although 6/14 of the host animals enrolled in the study experienced static or worsened ocular clinical signs following treatment, development of antiviral resistance is unlikely to be the underlying cause. At present, it is unknown why certain cats appear to respond to treatment with antiviral medications, but others do not.

While all 14 of the day 8 FHV-1 isolates were found to contain variants, there was no significant difference in the number of variants observed in isolates obtained from host animals receiving antiviral drugs or placebo. Studies of HSV-1 have determined that mixed viral populations are likely to exist both *in-vivo* and *in-vitro* and that the results of full genome sequencing is likely to represent an amalgamation of viral populations, rather than a single distinct isolate (37, 38). While this represents the most likely explanation for the apparent development of FHV-1 variants over a 7 day period, other possibilities include genuine genetic drift *in-vivo* or anomalies attributable to the known limitations of Illumina sequencing technology (39).

All three of the nonsynonymous variants detected were located in the FHV-1 ICP4 gene. This gene has previously been determined to act as a trans-acting factor (40). As such, it is possible that significant variants in this region may lead to functional alterations in promotion or inhibition of gene expression. All three of the day 8 isolates with a single non-synonymous variant in ICP4 developed a guanine to cytosine variant at CDS position 937. This corresponds with the ICP4 hypervariable repeat region, likely explaining this same finding occurring in three separate isolates. While the present study determined that these variants did not lead to functional antiviral medication resistance, it is possible that further *in-vivo* or *in-vitro* assessment could detect additional alterations in virulence. Based on the extremely limited number of non-synonymous variants detected, we speculate that this would be unlikely in the isolates included in the present study.

The plaque reduction assays performed on six of the isolates in the present study yielded half-maximal inhibitory concentrations within (cidofovir and penciclovir) or slightly higher than (ganciclovir) previously established ranges. Numerous reasons exist for these apparent discrepancies, including pipetting error, purity of the antiviral agents utilized *in-vitro* and cell culture media differences. These factors are unlikely to have influenced the results in the present study as these were all controlled for and standardized during the experiments. Although a wide range of FHV-1 IC_50_ values have been independently published for these drugs, ganciclovir consistently has low values, indicating relatively high FHV-1 inhibitory potency (7, 14).

The present study has several important limitations. The host animals from which the FHV-1 isolates were obtained received treatment or placebo for only 7 days and aside from upper respiratory tract disease were otherwise healthy. As noted above, HSV-1/2 isolates which are resistant to antiviral medications are generally obtained from immunocompromised individuals who are treated long-term. As such, it is unknown if immunocompromised feline hosts may be at increased risk of developing resistant FHV-1 isolates. The number of samples in the present study is low. The number of samples included was limited by the number of successful FHV-1 cultures obtained both before and after the treatment trial period; not all samples yielded viable virus or were found to be contaminated.

In conclusion, using example FHV-1 isolates obtained from animals treated with antiviral medications, we have detailed a method which can be utilized to assess the FHV-1 genome for mutation development and to assess the functional impact of mutations, if present. As the financial cost of full viral genome sequencing decreases and analysis expertise becomes more widely available, it is expected that the techniques outlined herein can be utilized in a clinical setting when resistance to antiviral drugs is suspected.

## Data availability statement

The datasets presented in this study can be found in online repositories. The names of the repository or repositories and accession number(s) can be found in the article or Supplementary material.

## Ethics statement

The animal study was reviewed and approved by Louisiana State University.

## Author contributions

AL conceptualized and designed the study. AL, NI, UE, MEM, MAM, and EPM organized the results and performed the *in-vitro* laboratory work outlined in this manuscript. AL and NI performed the genome variant analysis. C-CL performed the statistical analysis. AL wrote the first draft of the manuscript. All authors contributed to the article and approved the submitted version.

## Funding

This study was funded by a grant from the Morris Animal Foundation (D20FE-305); this manuscript has not been reviewed by the foundation and views expressed in this paper do not necessarily reflect the views of the foundation.

## Conflict of interest

The authors declare that the research was conducted in the absence of any commercial or financial relationships that could be construed as a potential conflict of interest.

## Publisher’s note

All claims expressed in this article are solely those of the authors and do not necessarily represent those of their affiliated organizations, or those of the publisher, the editors and the reviewers. Any product that may be evaluated in this article, or claim that may be made by its manufacturer, is not guaranteed or endorsed by the publisher.
